# Reply to ‘Comment on ‘Anthropometric measurements and survival after prostate cancer diagnosis”

**DOI:** 10.1038/s41416-018-0213-8

**Published:** 2018-07-31

**Authors:** Megan S. Farris, Kerry S. Courneya, Karen A. Kopciuk, S. Elizabeth McGregor, Christine M. Friedenreich

**Affiliations:** 1 Department of Cancer Epidemiology and Prevention Research, CancerControl Alberta, Alberta Health Services, Holy Cross Centre, Calgary, AB Canada; 2 Faculty of Kinesiology, Sport, and Recreation, University of Alberta, Edmonton, AB Canada; 3 Department of Oncology, Cumming School of Medicine, University of Calgary, Calgary, AB Canada; 4 Department of Mathematics and Statistics, University of Calgary, Calgary, AB Canada; 5 Population, Public & Indigenous Health, Alberta Health Services, Calgary, AB Canada; 6 Department of Community Health Sciences, Cumming School of Medicine, University of Calgary, Calgary, AB Canada

We thank Dal Maso et al.^1^ for their thought-provoking response to our recently published manuscript^2^ on the association between anthropometric measurements and survival after prostate cancer. We found no associations between anthropometry and prostate cancer prognosis and specifically, no associations with body mass index (BMI). Furthermore, we did not find any statistically significant interactions between smoking status and the association of BMI and survival, however, we did not present these analyses stratified by smoking status. Dal Maso et al.^1^ noted the potential for residual confounding by tobacco smoking status of this association and suggested that we consider this issue. Hence, we present here the additional analyses of the associations between anthropometric measures and prostate cancer outcomes stratified by smoking status.

To recap the main design aspects of our study, we had 987 incident, stage T2 or greater prostate cancer cases who were initially enrolled into a population-based case–control study between 1997 and 2000 who we followed up for all outcomes until 2017. BMI, along with other anthropometric measures were directly measured by interviewers shortly after prostate cancer diagnosis and 2–3-year postdiagnosis, along with several other lifestyle and prognostic factors. All-cause mortality and prostate cancer-specific mortality were determined for all participants on an ongoing basis through record linkage. To reanalyse our data by categories of smoking status and BMI, we performed a stratified analysis by smoking status (never, former, and current) in a Cox proportional hazards regression model. This analysis was completed for all-cause mortality and prostate cancer mortality at both time points: (1) shortly after diagnosis to within 6 months and (2) 2–3-year postdiagnosis. Smoking status was defined as: (1) never smokers (<100 cigarettes smoked over lifetime) and (2) former smokers (included both exoccasional and excurrent smokers who had stopped smoking for at least 1 month before the interview); current smokers (included occasional and regular smokers). All adjustments for possible confounding factors that we previously reported were done in these additional analyses. Tests for trend were also estimated for age-adjusted and multivariable adjusted models.

Table 1 presents the associations between BMI within 6-month postdiagnosis and all-cause mortality and prostate cancer-specific mortality by all smoking and BMI groups. The only statistically significant association found was an increased risk of prostate-specific mortality for obese men (BMI ≥ 30) relative to normal weight men (BMI < 25) who were former smokers at the time of diagnosis in the age-adjusted model (HR = 1.73, 95% CI: 1.02–2.92) with evidence of a trend (*p* = 0.007). This association, however, was attenuated in the multivariable adjusted model and no longer statistically significant (HR = 1.33, 95% CI: 0.76–2.31) and was not observed for all-cause mortality or for other categories of smokers. For the analyses done with the data collected at 2–3-year postdiagnosis, no statistically significant results were found for any of the associations between BMI and prostate cancer outcomes stratified by smoking status.

**Table 1:** Body mass index (kg/m^2^) in relation to all-cause mortality and prostate cancer-specific mortality stratified by smoking status in the Prostate Cancer Cohort Study in Alberta, Canada, 1997–2017

Smoking status by BMI category	All-cause mortality			Prostate cancer-specific mortality		
	All-cause deaths/cases	Age-adjusted HR (95% CI)	Multivariable adjusted HR (95% CI)^a^	Prostate cancer deaths/cases	Age-adjusted HR (95% CI)	Multivariable adjusted HR (95% CI)^b^
Baseline (within 6 months; *n* = 987)						
Never						
<25	35/60	1.0	1.0	16/60	1.0	1.0
25 ≤ 30	94/146	1.22 (0.83–1.80)	1.14 (0.76–1.71)	39/146	1.05 (0.59–1.88)	0.72 (0.38–1.34)
30+	41/69	1.36 (0.86–2.15)	1.37 (0.86–2.19)	22/69	1.29 (0.68–2.48)	1.12 (0.57–2.22)
Test for trend		0.18	0.19		0.42	0.66
Former						
<25	66/94	1.0	1.0	19/94	1.0	1.0
25 ≤ 30	179/289	0.89 (0.67–1.18)	0.77 (0.58–1.03)	60/289	0.99 (0.59–1.67)	0.76 (0.44–1.29)
30+	128/183	1.21 (0.90–1.64)	1.00 (0.74–1.37)	57/183	1.73 (1.02–2.92)	1.33 (0.76–2.31)
Test for trend		0.08	0.53		0.007	0.06
Current						
<25	47/59	1.0	1.0	18/59	1.0	1.0
25 ≤ 30	43/58	0.85 (0.56–1.29)	0.98 (0.62–1.55)	14/58	0.74 (0.37–1.50)	0.94 (0.42–2.06)
30+	23/29	1.30 (0.78–2.15)	1.38 (0.81–2.35)	7/29	0.90 (0.37–2.15)	1.11 (0.42–2.90)
Test for trend		0.54	0.31		0.64	0.89
In survivors during follow-up (2–3-year postdiagnosis; *n* = 829)						
Never						
<25	26/47	1.0	1.0	9/47	1.0	1.0
25 ≤ 30	69/114	1.07 (0.68–1.68)	1.00 (0.63–1.58)	31/114	1.41 (0.67–2.97)	1.08 (0.50–2.34)
30+	46/80	1.36 (0.84–2.21)	1.22 (0.73–2.03)	21/80	1.48 (0.67–3.23)	0.91 (0.39–2.13)
Test for trend		0.19	0.41		0.37	0.75
Former						
<25	48/64	1.0	1.0	13/64	1.0	1.0
25 ≤ 30	148/234	0.80 (0.57–1.11)	0.72 (0.51–1.01)	41/234	0.80 (0.43–1.49)	0.50 (0.25–0.96)
30+	104/175	0.84 (0.59–1.19)	0.73 (0.51–1.05)	44/175	1.20 (0.64–2.52)	0.99 (0.51–1.90)
Test for trend		0.51	0.19		0.23	0.22
Current						
<25	24/29	1.0	1.0	9/29	1.0	1.0
25 ≤ 30	39/53	0.80 (0.48–1.34)	0.95 (0.56–1.62)	10/53	0.54 (0.22–1.36)	0.58 (0.21–1.57)
30+	24/33	1.09 (0.60–1.96)	1.21 (0.66–2.23)	7/33	0.66 (0.24–1.83)	0.88 (0.29–2.65)
Test for trend		0.81	0.55		0.41	0.81

*HR* hazard ratio; *CI* confidence interval; *BMI* body mass index.

^a^Adjusted for age at diagnosis (continuous), stage of cancer (T2; T3/4; missing), prostatectomy (yes; no), hormone therapy (yes; no), radiation therapy (yes; no), PSA levels at diagnosis (<4; 4–10; >10–20; >20), postdiagnosis Charlson comorbidity score (continuous), total average alcohol consumption (continuous), smoking status at diagnosis (current; former; and never), Gleason score at diagnosis (<7; ≥7) and average frequency of going for a general check-up (yearly; every few years; occasionally; missing).

^b^Adjusted for age at diagnosis (continuous), stage of cancer (T2; T3/4; missing), prostatectomy (yes; no), hormone therapy (yes; no), radiation therapy (yes; no), PSA levels at diagnosis (<4; 4–10; >10–20; >20), postdiagnosis Charlson comorbidity score (continuous), total average alcohol consumption (continuous), region of residence (rural; urban), Gleason score at diagnosis (<7; ≥7) and average frequency of going for a general check-up (yearly; every few years; occasionally; missing)

Although there was a slightly increased risk of all-cause mortality amongst obese never smokers, as that found by Dal Maso et al.^1^ when compared with overweight or normal weight nonsmokers in our study, the risk estimates were not statistically significant and there was no evidence for trends across BMI categories. Indeed, the slightly elevated risks for all-cause mortality observed among obese never smokers were of similar magnitude to those found for obese current smokers compared to other BMI categories at both time points assessed in our study. Hence, it appears from our analysis, that there was no strong effect modification of the association between obesity and smoking on these prostate cancer outcomes in our study. The results from these stratified analyses need to be interpreted with caution given the small number of deaths in each analysis.

We agree with Dal Maso et al.^1^ that the association between obesity and prostate cancer survival is complex and requires careful consideration of the multiple and correlated factors that influence survival and that these need to be considered in future analyses of this topic.
